# Role of Biofilms in Children with Chronic Adenoiditis and Middle Ear Disease

**DOI:** 10.3390/jcm8050671

**Published:** 2019-05-13

**Authors:** Sara Torretta, Lorenzo Drago, Paola Marchisio, Tullio Ibba, Lorenzo Pignataro

**Affiliations:** 1 Fondazione IRCCS Ca’ Granda Ospedale Maggiore Policlinico, Policlinico of Milan, Via Francesco Sforza, 35, 20122 Milano, Italy; paola.marchisio@unimi.it (P.M.); Tullio.ibba@policlinico.mi.it (T.I.); lorenzo.pignataro@unimi.it (L.P.); 2 Department of Clinical Sciences and Community Health, University of Milan, 20122 Milan, Italy; 3 Clinical Chemistry and Microbiology Laboratory, IRCCS Galeazzi Institute and LITA Clinical Microbiology Laboratory, 20161 Milano, Italy; lorenzo.drago@unimi.it; 4 Department of Clinical Science, University of Milan, 20122 Milan, Italy; 5 Department of Pathophysiology and Transplantation, University of Milan, 20122 Milan, Italy

**Keywords:** Biofilm, adenoids, nasopharynx, upper respiratory tract infections, otitis

## Abstract

Chronic adenoiditis occurs frequently in children, and it is complicated by the subsequent development of recurrent or chronic middle ear diseases, such as recurrent acute otitis media, persistent otitis media with effusion and chronic otitis media, which may predispose a child to long-term functional sequalae and auditory impairment. Children with chronic adenoidal disease who fail to respond to traditional antibiotic therapy are usually candidates for surgery under general anaesthesia. It has been suggested that the ineffectiveness of antibiotic therapy in children with chronic adenoiditis is partially related to nasopharyngeal bacterial biofilms, which play a role in the development of chronic nasopharyngeal inflammation due to chronic adenoiditis, which is possibly associated with chronic or recurrent middle ear disease. This paper reviews the current evidence concerning the involvement of bacterial biofilms in the development of chronic adenoiditis and related middle ear infections in children.

## 1. Introduction

Chronic adenoiditis is frequent in children, mainly affecting those aged 3–7 years [1]. The adenoidal pads are located at the dome of the nasopharynx, and the enlargement of lymphoid tissue during childhood may contribute to the development of repeated nasopharyngeal infections, sleep disorders or middle ear infections after Eustachian tube orifice obstruction [2]. 

Bacterial biofilms may be involved in the pathogenesis of chronic adenoiditis, as they may be present throughout the nasopharyngeal mucosa, and particularly in the lateral portion of adenoidal pads extending towards the orifice of the Eustachian tube. 

Given the close anatomical and functional relationship between the nasopharynx and the middle ear (adenoidal pads may laterally extend to the ostium of the Eustachian tube), chronic adenoiditis is often complicated by the development of chronic or recurrent middle ear disease [3,4,5], which may manifest itself as persistent serous or mucous fluid in the middle ear (chronic otitis media with effusion, OME), repeated acute middle ear infections (recurrent acute otitis media, RAOM), or long-lasting middle ear suppuration through chronic perforation of the tympanic membrane (chronic suppurative otitis media, CSOM) [3,4,5]. Chronic OME is defined as documented as middle ear effusion without any sign of concomitant acute middle ear inflammation persisting for at least three months [4]; RAOM is defined as at least three episodes of acute otitis media within a period of six months, or more than four episodes in a period of 12 months [3]; and CSOM is defined as the presence of a long-lasting purulent ear discharge through a persistent perforation of the tympanic membrane [5]. Each of these conditions may significantly affect a patient’s quality of life and possibly predispose them to long-term functional sequelae and serious complications such as mastoiditis, meningitis, cerebral abscess, and lateral sinus thrombosis and auditory impairment [5]. A better understanding of the pathogenic mechanisms underlying these diseases would help to reduce their incidence and their related complications.

The bacteria mainly involved in the pathogenesis of acute and recurrent otitis media are the so-called otopathogens—*Haemophilus influenzae*, *Moraxella catarrhalis* and *Streptococcus pneumoniae*—but *Staphylococcus aureus* may also be involved, albeit less frequently [6,7]. Chronic otitis media is generally sustained by non-typeable *Haemophilus influenzae, Pseudomonas aeruginosa* and *Staphylococcus aureus* [8], and chronic nasopharyngitis and adenoiditis are due to the otopathogens *Staphylococcus aureus, Pseudomonas aeruginosa* and *Haemophilus parainfluenzae* [9].

It has been suggested that the lack of response to antibiotic therapy in children with chronic adenoiditis is partially attributable to the presence of nasopharyngeal bacterial biofilms, which may play a pathogenic role in the development of chronic nasopharyngeal inflammation due to chronic adenoiditis and are possibly associated with chronic or recurrent middle ear disease [9,10,11,12,13]. The failure of traditional antibiotic treatment is related to the resistance of bacterial biofilm, which is partially due to the physical barrier formed by the extracellular matrix, which blocks antibiotic diffusion within the biofilm, as well as some particular characteristics of the biofilms themselves [10], such as reduced bacterial replication in the inner layers and resistance mechanisms acquired as a result of the quorum-sensing process. Although various alternative conservative treatments have been tested (including manuka honey, baby shampoo, and hyaluronic acid- or acetylcysteine-based topical treatments), there is no evidence that they are effective in vivo. 

Given the largely ineffective nature of traditional antibiotic therapy, children with chronic adenoiditis (with or without middle ear involvement) are generally candidates for surgical treatment under general anaesthesia, including adenoidectomy and myringotomy, with or without the placement of a ventilation tube [14]. However, although the definitive treatment is surgical debridement, no clinical studies have investigated possible post-surgical changes in nasopharyngeal and middle ear biofilms.

This paper discusses current evidence concerning the involvement of bacterial biofilms in chronic adenoiditis and recurrent or chronic middle ear infections in children: English language papers concerning the possible role of bacterial biofilms in chronic adenoidal and middle ear disease that were published between 1 January 2007 and 1 January 2019 were selected after a MEDLINE search (accessed via PubMed). Separate systematic literature searches were made for chronic adenoiditis and middle ear disease (i.e., OME, RAOM and CSOM). Consideration was only given to fully accessible, original in vivo studies [9,11,12,15,16,17,18,19,20,21,22,23,24,25,26,27,28,29,30,31] published in peer-reviewed journals that specifically concerned the role of bacterial biofilm in chronic adenoiditis and recurrent/chronic middle ear disease in children. In vitro, ex vivo and animal studies, reviews and papers that did not clearly report the prevalence of bacterial biofilm involvement in their case series were excluded.

## 2. Bacterial Biofilms in Chronic Adenoiditis and Sampling Procedures

**Figure 1:**
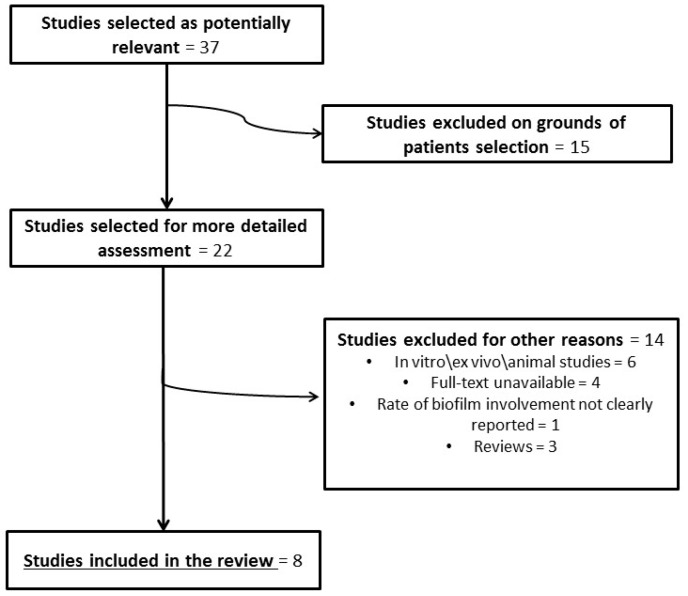
Flowchart of article selection (biofilms in chronic adenoiditis).

The literature search used the following terms: “biofilm AND nasopharyngitis”, “biofilm AND nasopharynx”, “biofilm AND adenoid”, and “biofilm AND adenoiditis”. This review was based on eight [9,11,12,15,16,17,18,19] of the 37 initially identified papers concerning chronic adenoiditis and included a total of 496 paediatric patients (Figure 1). 

The microbiological analyses of adenoid biopsies (collected during surgery) and nasopharyngeal swabs were made by means of spectrophotometry or other advanced microbiological techniques (scanning electron microscopy, fluorescence in situ hybridisation).

Analysis of the currently available literature suggests that the adenoids and surrounding nasopharyngeal mucosa are frequently covered by polymicrobial biofilm, and thus possibly act as an infectious focus in some cases. Bacterial biofilm can be found in up to 100% of nasopharyngeal samples taken from children with chronic nasopharyngeal infections. However, the reported prevalence rate varies widely (41%–100%) (Table 1) depending on the case series and the sampling and microbiological analysis techniques used [9,11,12,15,16,17,18,19]. The main bacteria involved were shown to be the otopathogens (*M. catarrhalis*, *H. influenzae*, *S. pneumoniae)*, *S. pyogenes*, *S. aureus* and *P. aeruginosa*. Table 1 shows the main characteristics of the selected case series and the related microbiological findings.

The microbiological analyses of nasopharyngeal specimens, which generally used spectrophotometry, scanning electron microscopy or fluorescence in situ hybridisation, showed that the main bacteria involved in nasopharyngeal biofilm production are the otopathogens (*H. influenzae*, *M. catarrhalis* and *S. pneumoniae)* [9,10,11,12,13], which are also responsible for middle ear infections.

The spectrophotometric method requires the incubation of bacterial cultures with appropriate growth medium in order to ensure biofilm development, after which the growth medium is discarded, each well is washed to eliminate any unbound bacteria, the adherent organisms are stained with crystal violet and any excess stain is rinsed away. Once the plates have been dried, the optical densities (ODs) of the stained adherent bacterial biofilms are measured using a microplate reader in order to assess bacterial adherence and biofilm formation [9].

Scanning electron microscopy of adenoidal specimens follows the method used by Hoa et al. [32]: the tissue samples are fixed, washed, dehydrated and coated for final imaging preparation and then imaged at a resolution of 1000× in order to study the biofilm architecture and analyse it using dedicated image analysis software.

The fluorescence in situ hybridisation of adenoidal samples with pathogen-specific 16S rRNA probes was described by Hoa et al. [32] as follows: specimens are fixed, washed and incubated, and then probed for *H. influenzae, S. pneumoniae, M. catarrhalis* and *S. aureus*, or using a universal eubacterial 16S rRNA probe.

There is no unanimous consensus concerning the preferred sampling technique as both adenoidal biopsies and nasopharyngeal swabs have been used, although the former has led to higher rates of positive findings. The well-known resistance of biofilms to mechanical injury reduces the negative predictive value of nasopharyngeal swabs in detecting biofilms [9], but the rate of adenoidal biofilm detection can also vary significantly in the case of bioptic specimens, probably because of the different methods used for fixation and histological processing. As the direction of mucociliary clearance leads to uneven biofilm distribution on the mucosal layer, the biofilm detection rate is higher in the caudal than the cephalic or middle sections [17]. Moreover, the traditional fixation method used during histological processing leads to the dispersion of sessile biofilms attached to the top of a fold on the nasopharyngeal surface, whereas the planktonic biofilm embedded in deep areas is preserved, because it is not subject to the actions of nasopharyngeal mucous flow [17].

Finally, the variability in detection rates of bacterial biofilm among patients with chronic adenoiditis may also be partially due to the different microbiological techniques used to detect them. Advanced but expensive electron microscopy techniques can more accurately evaluate the complex 3-dimensional structure of biofilm than the easier-to-use spectrophotometry technique, which can be used to quantify bacterial adhesion [9,10,11,12,13]. 

## 3. Nasopharyngeal Bacterial Biofilm: Is There A Correlation between Adenoiditis and Recurrent/Chronic Middle Ear Infections?

**Figure 2:**
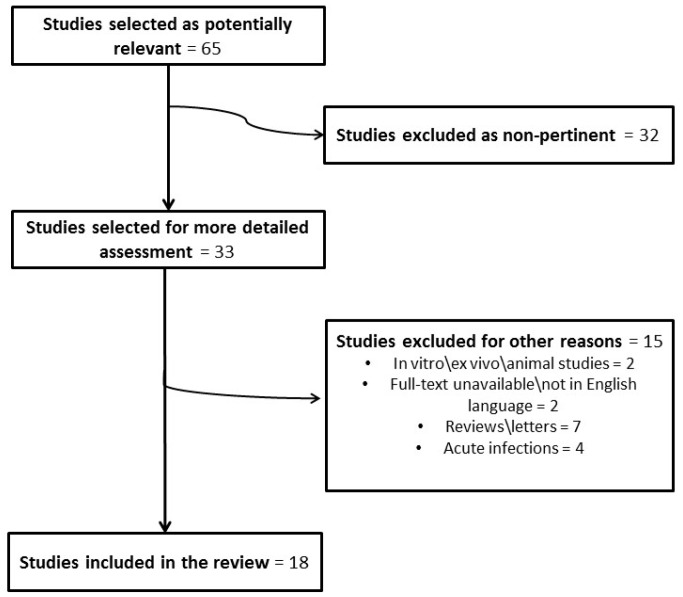
Flowchart of article selection (biofilms in recurrent/chronic middle ear disease).

The literature search was based on the terms “biofilm AND otitis”. Eighteen [20,21,22,23,24,25,26,27,28,29,30,31] of the 65 initially identified papers concerning recurrent/chronic middle ear disease with a total of 456 patients (Figure 2) were included.

The microbiological analyses of middle ear specimens (i.e., middle ear fluid/mucosa) conducted by spectrophotometry or other advanced microbiological techniques (scanning electron microscopy, fluorescence in situ hybridisation).

Biofilm involvement was detected in a variable proportion of patients with recurrent/chronic otitis media (0%–100%), depending on the case series and type of middle ear disease investigated: in 0%–100% of the adenoidal and/or middle ear samples collected from patients with OME; in 70%–80% of those taken from patients with RAOM; and in 83%–100% of those taken from patients with CSOM. The main bacteria involved were those listed above. Table 2 shows the main characteristics of the selected case series and the related microbiological findings.

The pathogenic role of bacterial biofilms in the development of chronic adenoiditis and middle ear disease still remains controversial, as, on one hand, it is supported by the detection of biofilms in a large prevalence of samples taken from the adenoids/nasopharynx of otitis-prone children (mainly children with RAOM and CSOM) [20,21,22,23,24,25,26,28,30,33,34,35,36,37,38,39,40,41] (Table 2). On the other hand, some papers show a reduced rate of nasopharyngeal/adenoidal biofilm involvement especially in children with OME [20,22,25,28]. Some electron microscopy studies have documented the massive distribution of biofilm on the surface of the adenoids of otitis-prone children who are candidates for adenoidectomy [12,13], and it has been reported that more than 93% of the adenoidal surface is occupied by biofilm in children with RAOM, whereas only 1% is involved in children undergoing surgery because of sleep disordered breathing.

The prevalence of adenoidal biofilms in children with a history of severe RAOM (defined as the presence of four acute otitis media episodes within a period of six months, or six in 12 months) is higher than that in children with sleep disordered breathing related to adenoidal hypertrophy but not to a history of middle ear infections [24]. Furthermore, the role of nasopharyngeal biofilms in favouring the development of non-severe RAOM was documented by Torretta et al. [13], who found that the rate of otopathogenic biofilms in the nasopharynxes of 58 children with non-severe RAOM was higher than that found in 55 healthy controls (about 41% vs. 14%). 

It has also been found that there is a variable prevalence of otopathogenic biofilms in specimens of middle ear mucosa/fluid taken from patients with persistent middle ear infections [34]; this suggests their involvement in the pathogenesis of persistent OME, only in some cases. Although OME was once considered bacteriologically sterile because of the difficulty of detecting pathogens by means of traditional culturing methods, the use of the polymerase chain reaction has led to the detection of bacterial DNA in the middle ear fluid of patients with persistent OME [36], and more recently, the discovery of bacterial mRNA in the middle ear mucosa of patients with OME has confirmed the bacterial hypothesis by documenting the presence of replicative bacteria [37]. The role of otopathogenic biofilms colonising the middle ear in the development of persistent OME is now considered, and scanning electron microscopy or confocal laser scanning microscopy studies of middle ear samples from children with OME have reported biofilm detection rates ranging from 40% to 92% [25,37]. However, the precise relationship between nasopharyngeal biofilms and OME is still unclear, and the published findings are conflicting. Saafan et al. [26] and Saylam et al. [23] described a greater prevalence of adenoidal biofilm in children with OME than in controls, whereas Hoa et al. [22] found lower prevalence of nasopharyngeal biofilm in children with persistent OME than in children with RAOM (28% vs. 99%).

Bacterial biofilms are also thought to be involved in the pathogenesis of chronic middle ear infections as they have been found in 43%–92% of middle ear specimens from patients with persistent perforations of the tympanic membrane leading to chronic middle ear suppuration (CSOM) [20,34,38,39] and in 75%–85% of specimens taken from patients with complicated disease sustained by the presence of dangerous chronic otitis media with cholesteatoma [20,33,39]. 

Regardless of whether otopathogenic nasopharyngeal and middle ear biofilms are involved in the development of chronic adenoiditis, RAOM or persistent OME, it is clear that the middle ear biofilms produced by *S. aureus* and *P. aeruginosa* play a pivotal role in CSOM [17] and chronic otitis media with cholesteatoma [20,34,35,38,39]. 

Finally, Moriyama et al. [40] hypothesised that bacterial biofilms are also responsible for the development of acute otitis media on the basis of the detection of biofilms produced by clinically non-typeable *H. influenzae* in patients with acute middle ear infections. This may explain the failure of traditional antibiotic treatment to cure patients with AOM, which may be related to the fact that otopathogens can produce biofilms rapidly in favourable environments [35]. However, this theory is still debated, as a study by Mizrahi et al. [41] failed to find any significant correlation between the presence of biofilm produced by non-typeable *H. influenza* and treatment failure or infectious recurrences in patients with AOM. These findings were supported by Osgood et al. [42], who suggested that the aerobic conditions occurring during AOM episodes may limit biofilm formation.

All of the above suggest a possible link between biofilm-related adenoiditis and chronic middle ear infections, whereas middle ear involvement is probably secondary to the colonisation of the middle ear mucosa by nasopharyngeal biofilm-producing otopathogens, which periodically spread in their planktonic forms during any acute infectious exacerbation. The subsequent migration of bacterial strains through an impaired Eustachian tube (due to the obstruction of its orifice by enlarged adenoidal pads) may lead to their adhesion to the middle ear mucosa and their subsequent sustenance of an independent chronic suppurative process whose periodic acute activation may be related to an autonomous middle ear biofilm. Despite this, given the high variability in detection rates and the fact that most previous studies did not evaluate the direct virulence of biofilm producers, no definitive conclusions about the pathogenic role of nasopharyngeal/adenoidal biofilm in the development of middle ear disease can be drawn.

## 4. Topographic Distribution of Bacterial Biofilms in the Nasopharynx.

**Figure 3:**
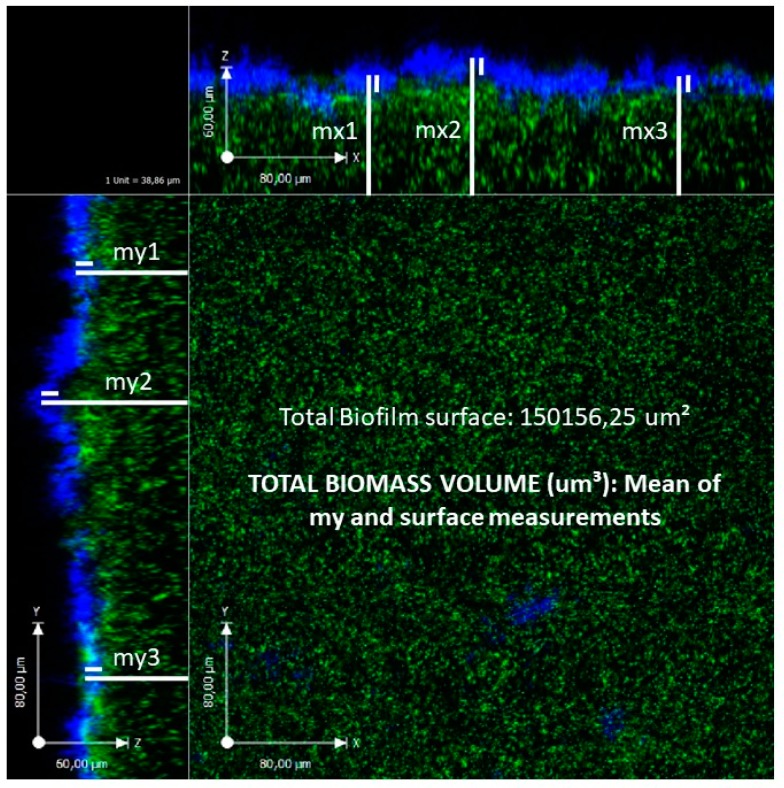
Total surface area of *S. aureus* biofilm after 48 hours (my = thickness measurement sides) as revealed by confocal laser scanning microscopy of an adenoidal specimen.

The different distribution of bacterial biofilms on the mucosal layer of adenoidal pads was investigated by Torretta et al. [11], who collected bioptic specimens for spectrophotometric analysis from the “nasopharyngeal dome” (i.e., the upper portion of the nasopharynx) and near the Eustachian tube orifice of children who were candidates for adenoidectomy because of chronic adenoiditis and persistent or chronic middle ear infections in order to investigate whether the two sites were different in terms of the prevalence of biofilm-producing otopathogens, the type of pathogens and the biofilm production capacity [11]. Biofilm-producing bacteria were detected in about 72% of the specimens from near the Eustachian tube orifice and about 53% of the specimens from the “nasopharyngeal dome”; *S. pneumoniae* and *M. catarrhalis* were more frequently isolated near the Eustachian tube orifice, and *S. aureus* was more frequently found at the “nasopharyngeal dome”, but the difference in strains was not statistically significant (Figure 3). However, the bacterial biofilms located near the Eustachian tube orifice were more frequently polymicrobial than those at the “nasopharyngeal dome”, thus suggesting that they were also more virulent [11]. 

It can therefore be speculated that chronically infected and enlarged adenoids (particularly when the adenoidal pads extend toward the Eustachian tube orifice) are sources of otopathogenic biofilms that periodically release planktonic species that are capable of colonising the Eustachian tube and then the middle ear mucosa. This suggests that surgical debridement during adenoidectomy needs to be as complete as possible (taking particular care to avoid any residues near the Eustachian tube orifice) in order to avoid biofilm survival in any persistent nasopharyngeal infectious focus. 

It has also been reported that nasopharyngeal biofilm-producing otopathogens are more prevalent in the nasopharynxes of young children with RAOM (but not chronic adenoiditis) than in healthy controls, which suggests that nasopharyngeal biofilms are independently involved in the development of recurrent middle ear infections regardless of the presence of adenoidal hypertrophy [13], which generally affects children aged > 3 years. 

## 5. Conclusions

Taken together, the current evidence documents the large prevalence of bacterial biofilms in adenoidal and middle ear samples taken from children with chronic adenoiditis with or without middle ear disease. This finding could suggest that otopathogenic nasopharyngeal biofilm is involved in the development and perpetuation of chronic adenoiditis. Subsequently, chronic biofilm-sustained adenoiditis may act as a possible risk factor to exacerbate adenoiditis-associated middle ear infections by predisposing some patients to developing independent and recalcitrant recurrent or chronic middle ear diseases such as RAOM, CSOM, and chronic otitis media associated with cholesteatoma. The involvement of bacterial nasopharyngeal/adenoidal biofilm in OME is questionable. In addition, in most studies, it is unclear whether biofilm producers are indeed highly and potentially virulent due to as over-producing toxins, having high biological fitness and/or being highly adhesive, or whether biofilm is solely generated as a result of repeated or chronic infections, when then act as dormant cells. Therefore, no definitive conclusions about the pathogenic role of nasopharyngeal/adenoidal biofilm in the development of middle ear disease can be drawn.

Surgical debridement is still the treatment of choice for eradicating nasopharyngeal biofilms, but scientific efforts should be made to test alternative non-surgical treatment strategies.

## 6. Key Concepts

1. Chronic adenoiditis frequently occurs in paediatric patients and is often complicated by the subsequent development of recurrent or chronic middle ear diseases.
2. The adenoids and surrounding nasopharynx may be sources of otopathogenic biofilms that periodically release planktonic species that are capable of colonising middle ear mucosa through an impaired Eustachian tube, thus predisposing individuals to the development of chronic nasopharyngeal and middle ear infections.
3. The main pathogens involved in nasopharyngeal biofilm production are the so-called otopathogens (*Haemophilus influenzae*, *Moraxella catarrhalis* and *Streptococcus pneumoniae*).
4. Given the specific topographic distribution of nasopharyngeal biofilm on adenoidal pads, adenoids should be carefully resected in the case of chronic nasopharyngeal infection, taking particular care to avoid any residues near the ostium of the Eustachian tube in order to avoid biofilm survival in any persistent nasopharyngeal infectious focus.
5. As nasopharyngeal biofilm-producing otopathogens seem to be also involved in the development of RAOM in young children without adenoidal hypertrophy, it can be speculated that nasopharyngeal biofilms are independently involved in the development of recurrent middle ear infections, regardless of the presence of adenoidal hypertrophy.
6. Future studies should investigate the potential virulence of biofilm-producers in the adenoids of otitis-prone children.

## Figures and Tables

**Table 1:** Main characteristics and related microbiological findings of the main studies of bacterial biofilm in chronic adenoiditis.

Authors; Year	No. of Patients; Samples	Mean Age ± SD, years	Analytical Technique	Prevalence of Bacterial Biofilm (%)	Prevalence of Isolated BPB
Galli et al. [15], 2007	15; 15	-	SEM	100	*H. influenzae* = 67% *S. pyogenese* = 7% Alpha-hemolytic streptococcus i = 13%
Al-Mazrou et al. [16], 2008	76; 76 (adenoids and tonsils)	5.7 ± 3.3	SEM	85	*Staphylococcus spp.* and *Streptococcus spp.*
Winther et al. [17], 2009	-; 9	-	PAS of Carnoy fluid and FISH	91	-
Torretta et al. [9], 2011	42; 84	7.0 ± 2.7	Spectrophotometry	BPB in 74 NPS and 69 B	*S. aureus* = 55% (NPS); 79% (B) *M. catarrhalis* = 19% (NPS); 21% (B) *H. influenzae* = 10% (NPS); 14% (B) *S. pneumoniae* = 35% (NPS); 10% (B) *P. aeruginosa* = 3% (NPS); 10% (B) *S. pyogenes* = 10% (NPS); 7% (B) *H. parainfluenzae* = 23% (NPS); 0% (B)
Torretta et al. [12], 2012	113; -	Median: 40 (range: 10–132), months	Spectrophotometry	BPO in 41 patients with recurrent middle ear infections and 14 controls	*M. catarrhalis* = 6 (patients); 34 (controls) *S. pneumoniae* = 17 (patients); 33 (controls) *S. pyogenes* = 6 (patients); 33 (controls) *H. influenzae*= 71 (patients); 0 (controls)
Torretta et al. [11], 2013	45; 135	Median: 7 (range: 4–13)	Spectrophotometry	BPB in 72 (ET); 53 (NPD)	*S. aureus* = 45% (ET); 50% (NPD) *S. pneumoniae* = 18% (ET); 12% (NPD) *M. catarrhalis* = 20% (ET); 12% (NPD) *S. pyogenes* = 9% (ET); 9% (NPD) *H. influenzae* = 3% (ET); 4% (NPD) Coagulase negative staphylococci = 3% (ET); 4% (NPD) *H. parainfluenzae* = 1% (ET); 4% (NPD) *P. fluorescens* = 1% (ET); 4% (NPD)
Kosikowska et al. [18], 2015	164; 328	Range: 2–5	Spectrophotometry	BP *Haemophilus* species in 97% of patients.	67% of *H. influenzae* samples were biofilm producers 56% of *H. parainfluenzae* samples were biofilm producers 86% of other *H.* spp samples were biofilm producers
Tsou et al. [19], 2015	32; -	Range: 4–13	Scanning electron microscopy	BP β-lactam-resistant *Haemophilus influenzae* type b more frequently detected in children with chronic adenoiditis than in those with adenoidal hypertrophy without infections.	

SD: standard deviation; B: biopsy; NPS: nasopharyngeal swab; SEM: scanning electron microscopy; PAS: periodic acid–Schiff stain; FISH: fluorescence in situ hybridisation; BPB: biofilm-producing bacteria; BPO: biofilm-producing otopathogens; BP: biofilm-producing; ET: near the Eustachian tube orifice; NPD: at the “nasopharyngeal dome” [11].

**Table 2:** Main characteristics and related microbiological findings of the main studies of bacterial biofilm in recurrent/chronic middle ear diseases.

Authors; Year	No. of Patients; Samples	Mean Age ± SD, Years	Disease	Analytical Technique	Prevalence of Bacterial Biofilm (%)	Prevalence of Isolated BPB
Homoe et al. [20], 2009	10; 13	Range: 2–15	CSOM OME	Peptide nucleic acid-FISH of MEM and MEF	83% 0%	*S. aureus* = 67% *S. maltophilia* = 17%
Zuliani et al. [21], 2009	68; 68	Range: 3 months–15 years	RAOM OSA	SEM of adenoidal mucosa	93% of adenoidal mucosa covered by biofilm in children with RAOM; 1% of adenoidal mucosa covered by biofilm in children with OSA	
Hoa et al. [22], 2010	30; 30	Range: 9 months–10 years	RAOM OME OSA	SEM of adenoidal mucosa	98% of adenoidal mucosa covered by biofilm in children with RAOM; 28% of adenoidal mucosa covered by biofilm in children with OME; and <1% of adenoidal mucosa covered by biofilm in children with OSA	
Saylam et al. [23], 2011	17; 17	7.5 ± 2.6	OME	SEM of adenoidal mucosa	100%	*-*
Nistico et al. [24], 2011	35; -	4.1 (range: 1–10)	COM OSA	CLSM and FISH of adenoidal mucosa	*H. influenzae, S. pneumoniae, S. aureus* polymicrobic biofilm in most samples	
Daniel et al. [25], 2012	42; 62	Median: 4.5 (range: 1–75)	OME	CLSM of MEM	49%	Coagulase-negative staphylococci = 3 *S. aureus* = 2 *S. pneumoniae* = 3 *Bacillus spp*. = 2 *M. catarrhalis* = 2 *Pseudomonas spp*. = 5 Other = 14
Saafan et al. [26], 2013	100; -	5.7 (range: 3–14)	OME	SEM and multiplex PCR of adenoidal mucosa and MEM	74% (adenoidal mucosa)	-
Thornton et al. [27], 2013	24; 38	Median: 17.9 (range: 9.7–36.0) months	RAOM	FISH on MEM	70%	*S. pneumoniae* = 73 *H. influenzae* = 65 *M. catarrhalis* = 27 *S. aureus* = 27 *P. aeruginosa* = 4
Szalmas et al. [28], 2013	59; -	5.1 (range: 3–11)	RAOM OME OSA	Hematoxylin-eosin and Gram staining of adenoidal mucosa	80% 0% 6%	*-*
Van Hoecke et al. [29], 2016	21; 34	3.3 (range: 1.1–6.6)	OME	FISH and CLSM of MEE	62% *H. influenza* biofilm	
Tawfik et al. [30], 2016	40; -	Range: 1–16	OME	SEM of adenoidal mucosa	100%	*-*
De la Torre et al. [31], 2018	10; 20	10 (range: 6–17)	Cholesteatoma	CLSM of cholesteatoma	100%	*-*

SD: standard deviation; BPB: biofilm-producing bacteria; SEM: scanning electron microscopy; CLSM: confocal laser scanning microscopy; FISH: fluorescence in situ hybridisation; PCR: polymerase-chain reaction; MEM: middle ear mucosa; MEF: middle ear fluid; OME: chronic otitis media; RAOM: recurrent acute otitis media; OSA: obstructive sleep apnoea; COM: chronic otitis media (unless otherwise specified); CSOM: chronic suppurative otitis media.
